# Unusual Neurological Complications in a Patient With Monkeypox: A Case Report

**DOI:** 10.7759/cureus.58479

**Published:** 2024-04-17

**Authors:** Qassem Hammad, Zahra Mansour Alalshaikh, Zeidan A Zeidan, Syed Islam, Albishi Haya

**Affiliations:** 1 Internal Medicine, King Salman Hospital, Riyadh, SAU; 2 Infectious Disease, King Salman Hospital, Riyadh, SAU; 3 Infection Control, King Salman Hospital, Riyadh, SAU; 4 Radiology, King Salman Hospital, Riyadh, SAU

**Keywords:** skin rash, pulse steroid, encephalomyelitis, monkeypox, case report

## Abstract

Monkeypox is a zoonotic disease caused by an enveloped single-stranded DNA virus that belongs to the Poxviridae family. It was first identified in humans in the 1970s. In 2022, a monkeypox outbreak spread extensively outside of endemic countries. Monkeypox infection begins with the prodromal symptoms of fever, myalgia, and lethargy, followed by the development of a characteristic maculopapular rash. In most cases, the illness is self-limiting. However, severe cases can lead to devastating neurological complications, such as encephalitis. Here, we present the case of a 31-year-old male patient with monkeypox who developed encephalomyelitis and exhibited complete neurological recovery upon treatment with pulse steroid and intravenous immunoglobulin.

## Introduction

Monkeypox is a zoonotic disease caused by an enveloped single-stranded DNA virus in the Poxviridae family. Monkeypox virus was discovered during an outbreak in 1958 in Denmark, Copenhagen [1]. It was first identified in humans in the 1970s in the Democratic Republic of the Congo [2]. Monkeypox virus infects various types of mammals. Although the specific animal species that serve as the definitive host and reservoir for the virus remains unknown, various rodent species, including African squirrels and Gambian pouched rats, have also been suggested [1]. Monkeys are considered intermediate hosts [3]. In 2022, an outbreak of monkeypox spread outside of the endemic countries in central and west Africa [4].

Human-to-human spread of the virus typically occurs through direct contact with the monkeypox rash, scabs, or bodily fluids or prolonged contact with respiratory secretions from infected persons. The virus may also spread through contact with fomites (surfaces that come into contact with the virus). It is not known whether monkeypox can be transmitted through semen or vaginal secretions, and the mechanisms for this potential form of transmission are not entirely understood [5].

The mean incubation period is 8.5 days (with the 5th to 95th percentiles ranging from 4.2 to 17.3 days). It is worth noting that the incubation period might vary depending on the transmission route [6]. It starts with a prodrome, which includes symptoms such as fever, myalgia, and lethargy, followed by the development of a characteristic maculopapular rash. These lesions can occur and spread in a centrifugal pattern [7] on the genitals, skin, and the inside of the mouth and throat. They typically progress through different stages, including macules (flat discolored spots), papules (raised bumps), vesicles (fluid-filled blisters), pustules (pustular lesions), and crusts [8].

In most cases, monkeypox is a self-limiting illness; however, severe cases have been reported. The Central African clade is associated with a more severe form of monkeypox compared to the West African clade. The mortality rate in the Central African clade was 10.6%, in contrast to the West African clade of 3.6% [9]. A rare but serious complication is encephalitis in fewer than 1% of cases, which leads to neurological symptoms such as severe headaches, confusion, seizures, and coma [10]. Cases of monkeypox resulting in fatal encephalitis are exceedingly rare with only a few cases reported [11,12].

Therefore, clinicians must be aware of these potential complications and consider them in the evaluation and management of patients with monkeypox, particularly in patients with severe cases or neurological symptoms. Early recognition and appropriate medical intervention are essential to improve outcomes and prevent life-threatening complications.

## Case presentation

A 31-year-old male presented to our emergency department (ED) with a vesiculopapular rash on both arms and genitals that had appeared seven days prior. Two days before the ED visit (during the second week of the infection), the patient had developed symptoms of worsening weakness and numbness in both feet. Within two days, the weakness rapidly progressed to complete paralysis, and the patient developed urinary retention, difficulty swallowing, right-sided facial drop, and disorientation. He became agitated during the visit and required physical restraint and sedation.

The patient was a heterosexual male and reported having unprotected sexual intercourse with a female who had the same skin rash but no neurological symptoms five days before symptom onset. There was no family history of a genetic disorder. He was in a good psychosocial state and did not report any chronic illness or previous similar condition.

Clinically, he was somnolent and showed right-sided peripheral facial weakness, dysarthria, paraplegia (power: 0/5), and absent bilateral reflex in both lower limbs. In addition, he exhibited impaired dull, pin-prick, pain, temperature, vibration, and proprioceptive sensations at the T11 spinal segment level. Bilateral pupil reflexes were normal. There was no upper extremity weakness or loss of sensation. Meningeal signs were not observed. Moreover, Kernig’s sign and Brudzinski’s sign were negative, and there was no neck stiffness.

Laboratory tests on admission showed a mildly elevated white blood cell count and an elevated erythrocyte sedimentation rate with normal C-reactive protein levels. Renal and liver function tests were normal. Human immunodeficiency virus antibodies, hepatitis B surface antigen, hepatitis B virus core antigen, hepatitis B surface antibodies, and hepatitis C antibodies were unremarkable. Vitamin B12 and folate were normal.

Monkeypox virus DNA was detected by quantitative real-time polymerase chain reaction (qRT-PCR) of cutaneous lesion swabs (diagnostic test). The lumbar puncture showed normal opening pressure, clear cerebrospinal fluid (CSF), mildly elevated protein, and lymphocytic pleocytosis (Table 1). Real-time PCR of the CSF and transverse myelitis laboratory workup were not available.

**Table 1:** Results of CSF evaluation. CSF = cerebrospinal fluid; TB = tuberculosis; PCR = polymerase chain reaction

Test	Result	Reference
CSF glucose	71.28 mg/dL	50–75 mg/dL
Blood glucose	75 mg/dL	70–100 mg/dL
CSF proteins	68 mg/dL	6–8.5 mg/dL
Leukocytes	25 cells/mm^3^	<5 cells/mm^3^
Lymphocytes	68%	
Polymorphic cells	36%	
Gram staining	Negative	Negative
Bacterial culture	Negative	Negative
TB PCR	Negative	Negative
TB culture	Negative	Negative

**Figure 1:**
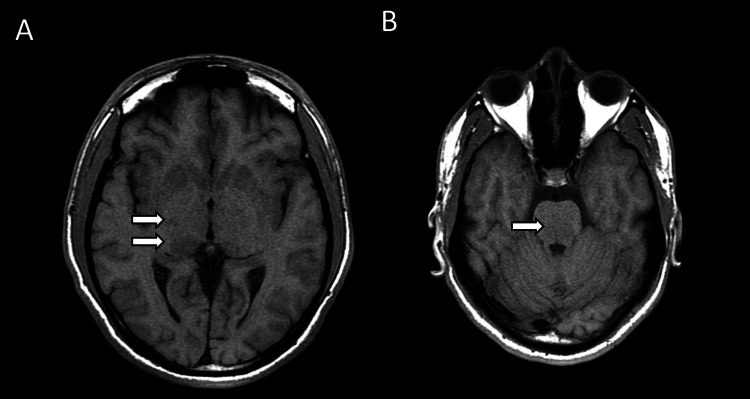
Axial T1-weighted sequence showing multiple hypointense signals in the (A) basal ganglia, thalamus, and (B) brainstem.

**Figure 2:**
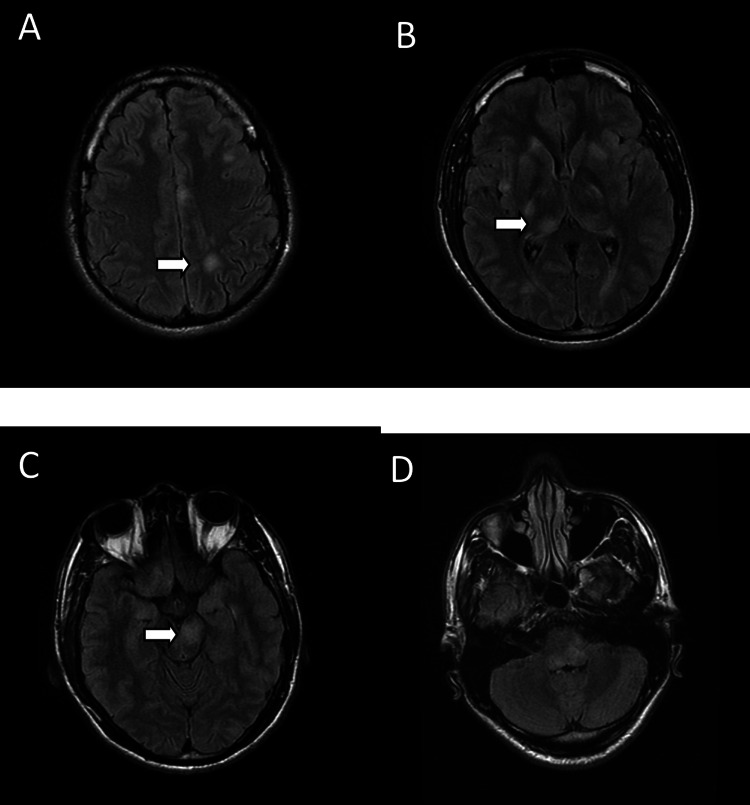
(A) Axial fluid-attenuated inversion recovery images show multiple, rounded, ill-defined hyperintense signals in the left frontal, parietal, and occipital lobes in the cortical and subcortical regions. Hyperintense signals are seen in (B) the bilateral basal ganglia, thalamus, right insular cortex, medial temporal lobe, and (C and D) brainstem.

**Figure 3:**
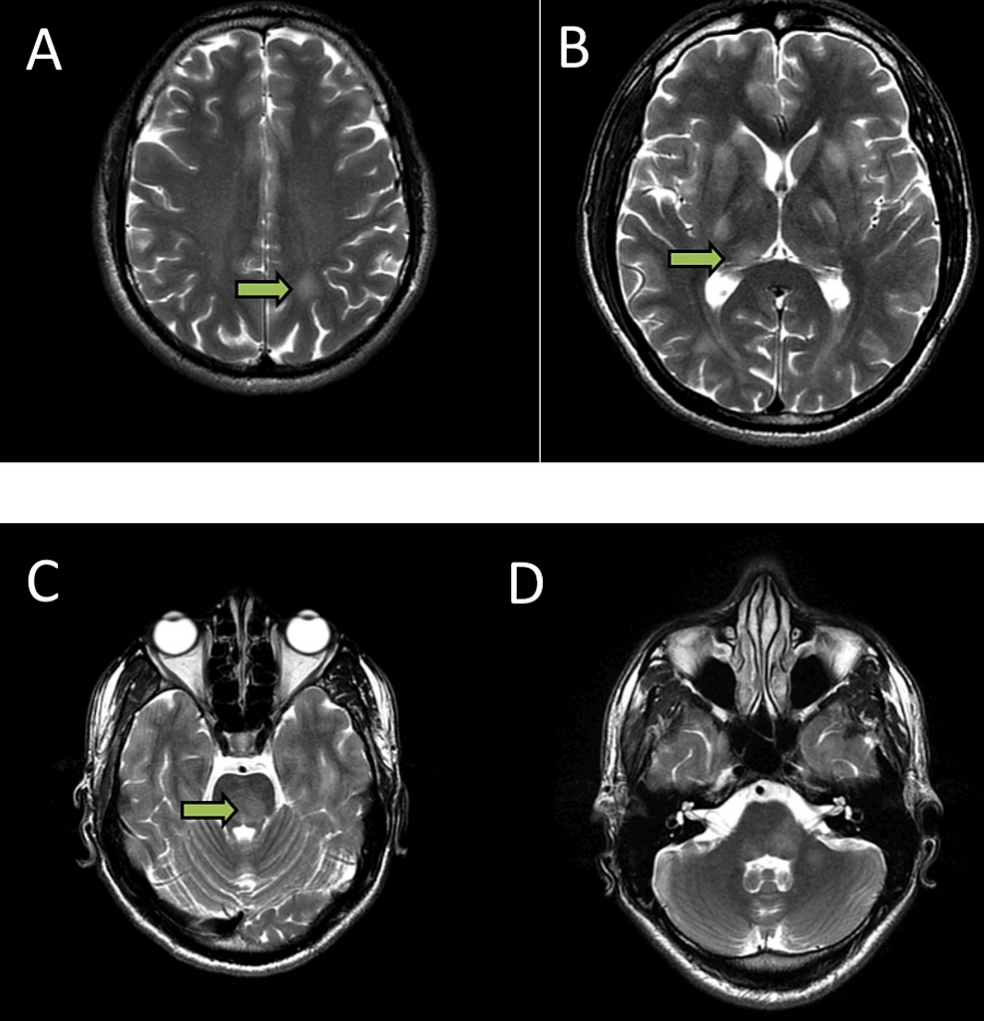
(A) Axial T2 sequence showing hyperintense signals in the left frontal, parietal, and occipital lobes in the cortical and subcortical region. (B) Hyperintense signals are seen in bilateral basal ganglia, thalamus, right insular cortex, (C and D) medial temporal lobe, and brainstem.

**Figure 4:**
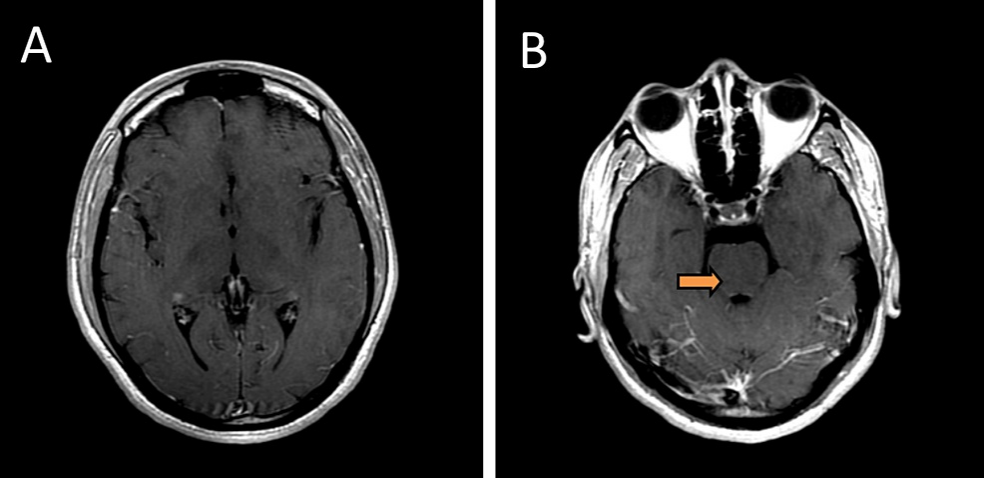
(A and B) Post-contrast T1 magnetic resonance imaging showing very subtle enhancement in the brainstem.

**Figure 5:**
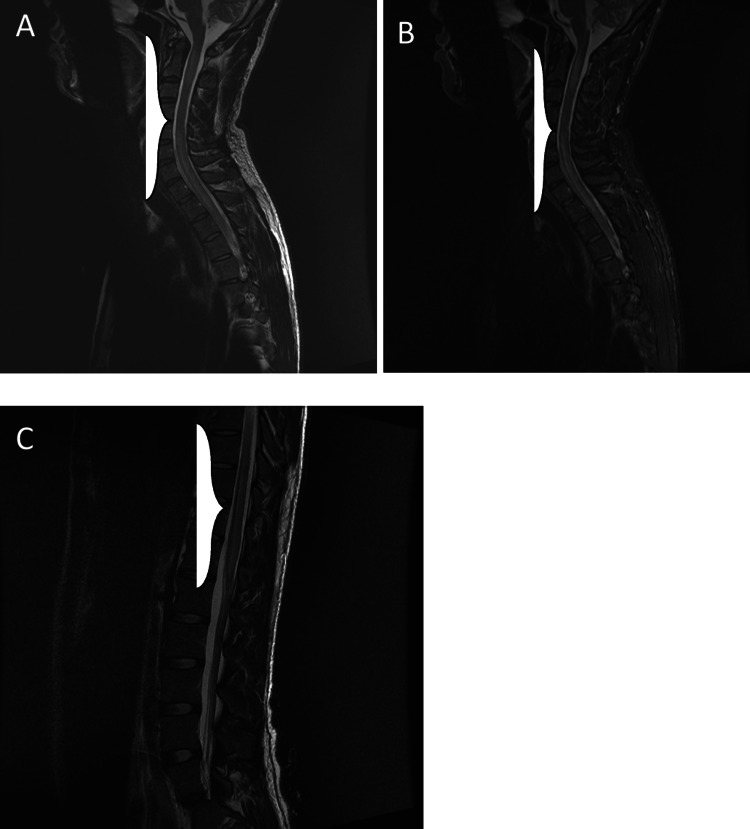
(A and B) Sagittal T2 and short tau inversion recovery of the cervical, upper, and lower thoracic and lumbar spine. Long-segment hyperintense signals are seen in the cervical and upper thoracic spinal cord with mild cord swelling in the upper cervical spine.

**Figure 6:**
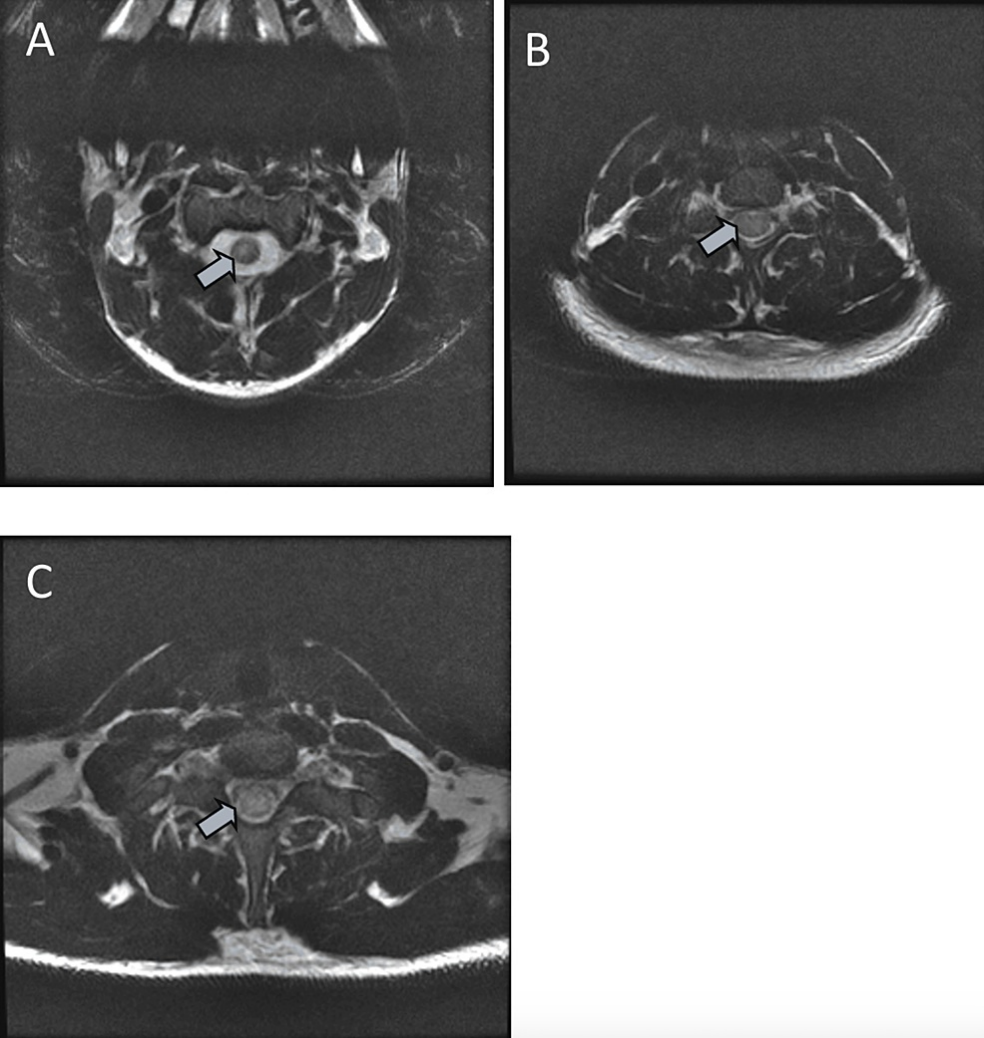
(A-C) More than two-thirds cross-sectional area of hyperintense signals is seen in the spinal cord in the cervical and upper thoracic region.

**Figure 7:**
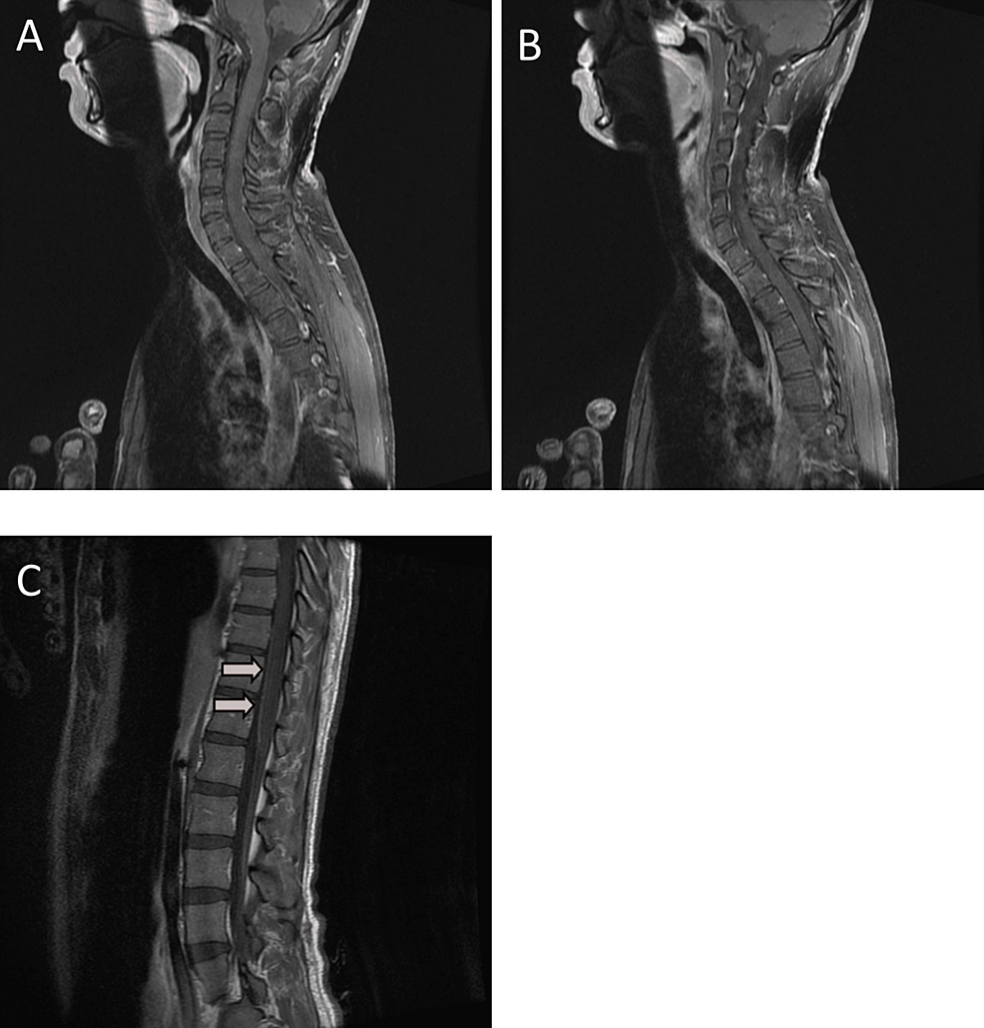
(A-C) Sagittal T1 post-contrast fat-suppressed sequence. No enhancement is seen in the spinal cord. Increased meningeal enhancement is noted in both the anterior and posterior margins of the spinal cord.

Magnetic resonance imaging (MRI) of the brain and spine (T2 and fluid-attenuated inversion recovery (FLAIR) sequences) revealed multiple lesions in cortical and subcortical regions in both cerebral hemispheres. Hyperintense signals were also observed in the deep gray matter nuclei and the brainstem. In the spine, long-segment hyperintense T2 and FLAIR signals were noted in the cervical and upper thoracic spinal cord and occupied more than two-thirds of the cross-sectional area of the spinal cord. In post-contrast images, no enhancing lesions were observed in the spinal cord or brain; however, increased meningeal enhancement was identified along the anterior and posterior margins of the spinal cord. In the MRI differential diagnosis of inflammatory processes, myelitis and demyelinating disease were proposed. See Figures 1-7 for detailed findings.

The patient was initially managed (on day one) with five doses of intravenous immunoglobulin (IVIG) (0.2 g/kg QD) (Octagam from Octapharma company), followed by antivirals (brincidofovir 200 mg PO OD in two doses, eight days apart on day three). As there was no response to previous treatment, a decision was made to start systemic corticosteroids (methylprednisolone 1 g QD) for five days. Blood, urine, and sputum cultures were sent before administering steroids and were negative. No side effects were noted post-intervention.

We did not opt for plasma exchange because it would mitigate the effects of the IVIG. At a four-week follow-up, the clinical condition of the patient had improved, with complete resolution of cutaneous lesions. The patient showed significant improvements in communication, urinary control, and lower limb power (5/5).

## Discussion

This case report details an atypical manifestation of monkeypox complicated by encephalomyelitis in a previously healthy 31-year-old male. The patient presented with a vesiculopapular rash persisting for seven days on both arms and genitals. By the second week of infection, symptoms indicative of encephalomyelitis emerged, including numbness in the lower extremities, rapidly progressing weakness leading to complete paralysis, urinary retention, and altered sensorium. This disease progression mirrored that of a previously documented case by Marín-Medina et al., where encephalomyelitis symptoms emerged during the second week of infection [13]. According to a systematic review and meta-analysis by Badenochet et al., only 2% of monkeypox patients develop encephalitis [14].

Our laboratory and imaging findings closely resembled those reported in the literature. Monkeypox virus DNA was detected via qRT-PCR of cutaneous lesion swabs. Laboratory tests indicated mildly elevated white blood cell counts and erythrocyte sedimentation rates, consistent with findings reported by Yadav et al. [15]. MRI revealed spinal cord lesions resembling those observed in a case reported by Cole et al. [16]. CSF analysis showed elevated protein levels and normal lymphocyte levels, consistent with findings reported by Marín‑Medina et al. [13].

Treatment involved IVIG, the antiviral tecovirimat, and systemic corticosteroids. After four weeks, the patient showed significant clinical improvement, including complete resolution of cutaneous lesions, improved cranial nerve function, and increased limb strength. He was discharged for outpatient rehabilitation therapy and fully recovered two months post-discharge.

Management strategies for similar cases of monkeypox with encephalomyelitis vary. Studies of patients receiving the same treatment regimen have reported subsequent improvement [13]. Patients who developed monkeypox-related encephalomyelitis before Food and Drug Administration approval of tecovirimat and did not receive antivirals also improved [16].

While the routine use of IVIG or corticosteroids in monkeypox management is not routinely recommended [17], decisions regarding their use should be based on clinical judgment, symptom severity, and individual patient factors.

## Conclusions

We report a rare case involving a 31-year-old male patient who presented with paraplegia and a skin rash. The presence of the monkeypox virus was confirmed through DNA qRT-PCR testing of swabs taken from cutaneous lesions. Additionally, the patient developed encephalomyelitis. However, following treatment with brincidofovir, pulse steroid therapy, and IVIG, the patient experienced complete neurological recovery.
